# Monitoring of Corroded and Loosened Bolts in Steel Structures via Deep Learning and Hough Transforms

**DOI:** 10.3390/s20236888

**Published:** 2020-12-02

**Authors:** Quoc-Bao Ta, Jeong-Tae Kim

**Affiliations:** Department of Ocean Engineering, Pukyong National University, Nam-gu, Busan 48513, Korea; qb.tabao@gmail.com

**Keywords:** deep learning, image-based monitoring, regional convolutional neural network, Hough line transform, bolt corrosion, bolt-loosening, bolted connection, steel structure

## Abstract

In this study, a regional convolutional neural network (RCNN)-based deep learning and Hough line transform (HLT) algorithm are applied to monitor corroded and loosened bolts in steel structures. The monitoring goals are to detect rusted bolts distinguished from non-corroded ones and also to estimate bolt-loosening angles of the identified bolts. The following approaches are performed to achieve the goals. Firstly, a RCNN-based autonomous bolt detection scheme is designed to identify corroded and clean bolts in a captured image. Secondly, a HLT-based image processing algorithm is designed to estimate rotational angles (i.e., bolt-loosening) of cropped bolts. Finally, the accuracy of the proposed framework is experimentally evaluated under various capture distances, perspective distortions, and light intensities. The lab-scale monitoring results indicate that the suggested method accurately acquires rusted bolts for images captured under perspective distortion angles less than 15° and light intensities larger than 63 lux.

## 1. Introduction

Bolts serve to connect structural components and to maintain the load-bearing performance of a steel structure. As objects exposed to severe environmental factors like moisture and air pollutants, poorly maintained bolts become vulnerable to local failure due to progressive corrosion [1,2]. Also, bolted connections may experience mechanical shocks during construction and vibrational shivering in service, leading to bolt rotations. Therefore, robust monitoring techniques should be implemented to detect damage in bolted connections at an early stage and to secure the structural integrity under multiple hazard conditions.

Many structural health monitoring (SHM) techniques have been developed to replace traditional time-costly visual inspections [3,4,5,6]. Damage in bolted connections has been tracked by using contact sensor-based techniques which include acoustic-based methods utilizing ultrasonic time of flight through the bolt [7], wavelet analysis-based methods using accelerometer sensors to measure the signal change of before and after the damaged bolts [8], piezoelectric active sensing methods using ultrasonic energy passing through the contact interface, and electromechanical impedance methods utilizing the variation of impedance caused by bolt-loosening [1,9]. However, those contact sensor-based SHM approaches require high-precision instrumentation, together with built-in algorithms, to compensate for environmental variation effects [10,11]. To deal with any structural connection containing numerous bolts, those approaches require many sensors and expensive measurement instruments.

Recently, vision-based SHM has been emerged as preference, compared to the contact sensor-based methods. The advantages of the approach include non-contact sensing, low cost, handy setup and operation. By utilizing intuitive image data captured by digital cameras, vision-based approaches can efficiently acquire structural information at diverse local points. In addition, the damage-sensitive image features are immune to changes in environmental conditions, thus minimizing uncertainties that could lead to false warnings due to temperature or humidity.

To date, a few research attempts have been made to develop vision-based SHM methods for bolted connections [12,13,14,15,16]. Park, et al. [12] designed a binary image-based bolt angle detection approach using image processing tools like Hough line transform [17] and canny edge detector [18]. Their method could accurately detect a minor bolt rotation of 4° [12]. Cha, et al. [13] and Ramana, et al. [16] proposed image processing algorithms using support vector machines together with a Viola-Jones algorithm to automate the bolt-loosening detection process. Their methods could detect bolt-loosening with the exposed thread length equal to or larger than 5 mm induced by bolt rotation.

Recently, deep learning technology has produced a variety of computer vision-based SHM applications. Deep learning based on a convolutional neural network (CNN) has been considered as an efficient approach for object identification [19]. Zhao, et al. [20] used a CNN algorithm to detect the angle of a non-corroded bolt by monitoring the shift of a pre-marked sign on the bolt. The performance of the method should be evaluated for practical conditions of real bolts which might have weird signs (i.e., rust or defect) on detected surface areas. Atha, et al. [21] proposed a CNN-based deep learning model for corrosion assessment of metallic surface, evaluating effects of various color spaces, sliding window sizes, and CNN architectures on the computation time. Moreover, the CNN-based deep learning model could localize other damage features by adding more types of damage to training data along with using the larger perceptron capacity [19]. Cha, et al. [22] proposed a faster region-based CNN method to detect multiple damage types such as steel corrosion and concrete crack; the method, however, only tests the damage at one physic light condition. Huynh, et al. [23] proposed a two-phase method which consists of RCNN-based bolt identification and graphical image processing-based bolt angle estimation. Their method could accurately estimate small bolt-loosening, but with limited applicability for non-corroded bolts.

Despite the above research efforts, there exists a need to improve vision-based methods for accurate detection of complex damage types such as corrosion and bolt-loosening in bolted connections. To this end, this paper presents a regional convolutional neural network (RCNN)-based deep learning and Hough line transform (HLT) algorithm to autonomously monitor bolt corrosion and loosening in steel structures. Two specific goals are to detect rusted bolts distinguished from non-corroded clean ones and also to estimate bolt-loosening angles of the identified bolts. The following approaches are performed to achieve the goals. Firstly, a RCNN-based deep learning model is designed to detect bolts and to distinguish corroded bolts from clean ones. Next, the HLT-based algorithm is employed to estimate the rotational angle of cropped bolt images in the target connection. Finally, the accuracy of the proposed framework is experimentally evaluated under various capturing distances, perspective distortions, and light intensities.

## 2. Image-Based Monitoring of Corroded Bolts

### 2.1. Workflow of Bolt Detection and Bolt Angle Estimation

**Figure 1:**
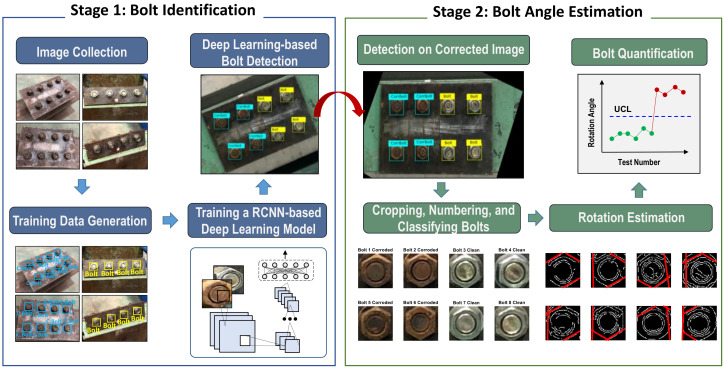
Overview of the proposed framework.

A framework is designed to autonomously classify clean and corroded bolts in steel joints and also to estimate rotational angles of them. As described in Figure 1, the overall process of the framework consists of two primary stages: deep learning-based identification of corroded bolt (Stage1) and binary image-based bolt angle estimation (Stage 2).

In Stage 1, a RCNN-based deep learning model is designed to recognize and localize clean and corroded bolts in a target image. The task consists of four steps: (1) to collect target bolt connection images which contain clean and corroded bolts, (2) to generate training data by assigning correct labels for corresponding bolts, (3) to train a RCNN-based deep learning model by computing bolt features, and (4) to classify clean and corroded bolts based on the trained model.

In Stage 2, a binary image-based model is designed to estimate the rotation angle of each detected bolt. Firstly, the detected bolt image is adjusted by correcting the perspective distortion. It is cropped into a single image for estimating bolt angles. Secondly, all cropped bolts are re-allocated into four damage scenarios: clean and no-rotation (S1), corrosion and no-rotation (S2), clean and rotation (S3), corrosion and rotation (S4). Thirdly, bolt angles are estimated for the scenarios S3 and S4 by using the HLT algorithm. Finally, the estimated bolt angles are statistically quantified to make the decision on their bolt-loosening severities.

### 2.2. Deep Learning-Based Bolt Detector

#### 2.2.1. RCNN-based Classification of Clean and Corroded Bolts

**Figure 2:**
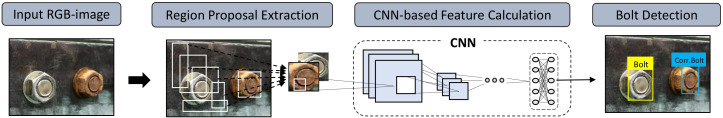
The general approach of RCNN-based bolt detector.

The RCNN-based deep learning method proposed by Girshick, et al. [24] is one of the most useful methods for object detection. The general approach of a bolt detector based on the RCNN-based method is illustrated in Figure 2. Firstly, the RGB-input image is taken by an iPhone X’s camera. Secondly, a selective search algorithm [25] is applied to identify candidate objects by creating approximately 2000 regions per second, which automatically crop potential objects (i.e., the region of interest (RoI)) and then input them into the CNN-based classifier. Thirdly, each of the cropped RoI is reshaped by warping to a fixed size of the image and then forwarded to the CNN-based feature calculation. Finally, the RoI is classified and marked by labeling non-corroded bolt or corroded bolt in the image.

**Figure 3:**
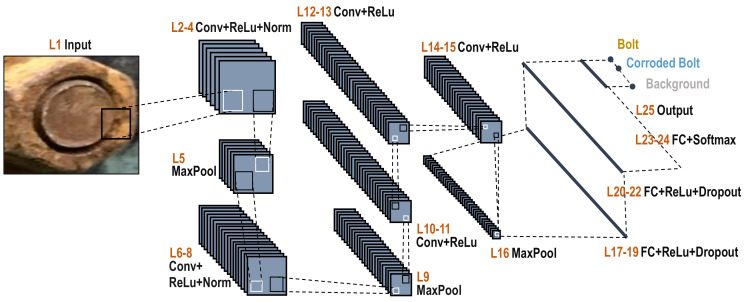
The schematic of a CNN-based bolt classifier.

The schematic of the CNN-based bolt classifier is depicted in Figure 3. The AlexNet-based CNN architecture, proposed by Krizhevsky, et al. [26], is adopted to fine-tune for extracting bolt features. Detailed description (i.e., layer, size, operator, filter size, etc.) of the CNN architecture are detailed in Table 1. The CNN-based bolt classifier is to identify three inferences which are clean bolt, corroded bolt, and background. The CNN model consists of an input layer, five convolutional layers, three fully connected (FC) layers, seven rectified linear unit (ReLU) layers, two normalization operation (norm) layers, two dropout layers, three MaxPooling layers, a softmax layer, and an output layer.

#### 2.2.2. Training of an RCNN-Based Bolt Detector

**Figure 4:**
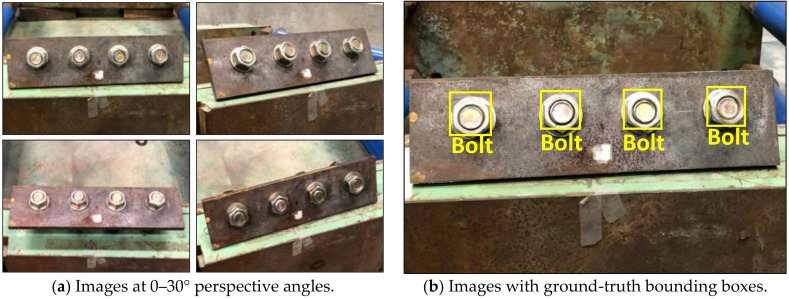
Training data of non-corroded bolts in a steel plate 1 for RCNN-based bolt detector.

Two separate samples of clean (non-corroded) and corroded bolts are produced to build a database for training a RCNN-based bolt detector. As shown in Figure 4, four training images of clean bolts were simulated in a steel splice plate of 310 × 100 × 10 mm (steel plate 1). Bolts were equally distanced by 70 mm. The images were captured at various vertical and horizontal perspective angles of 0–30° (see Figure 4a). As shown in Figure 4b, four ground-truth bounding boxes were selected as the non-corroded bolt ‘bolt’.

**Figure 5:**
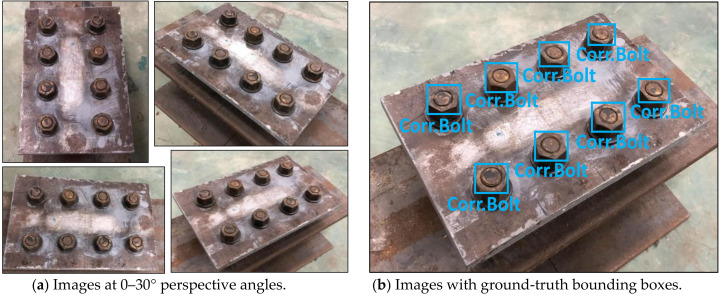
Training data of corroded bolts in a steel plate 2 for RCNN-based bolt detector.

As shown in Figure 5, eight training images of corroded bolts were simulated in a steel splice plate of 310 × 200 × 10 mm (plate 2). Bolts were equally distanced by 70 mm horizontally and 100 mm vertically. As shown in Figure 5a, the images were captured at different perspective angles of 0–30°. As shown in Figure 5b, eight ground-truth bounding boxes were selected as the corroded bolt ‘Corr.Bolt’. All of non-corroded and corroded bolts were captured at the distance of 1.0–1.5 m. The light intensity was roughly 154–350 lux. In total 419 images were taken for non-corroded bolts (189 images) and corroded bolts (230 images).

The RCNN-based bolt detector uses a transfer learning using AlexNet [26], which allows a faster learning of bolt image features. By pre-training the deep learning model with a large number of image sets, a fine-tuning approach could be applied to efficiently train the model with relatively small data sets. In order to produce a pre-trained AlexNet CNN model with 419 bolted connection images, a back-propagation algorithm named stochastic gradient descent with momentum (SGDM) is employed using a learning rate of 0.000001 and a mini-batch size of 128. For the training process of the 419 images, the RCNN-based bolt detector with 50 epochs took roughly 75 min using a desktop computer system (GPU: GTX 1080 Ti 11G, CPU: Intel i7-8700K 3.7 GHz, RAM: 32 GB).

**Figure 6:**
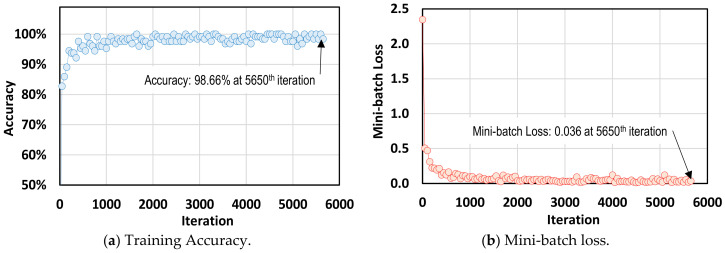
Training process of RCNN-based bolt detector.

Figure 6 shows the training process of the RCNN-based bolt detector. After training with 5650 iterations (50 epochs), the accuracy reached up to 98.66% for positive training samples with the bounding box overlap ratios greater than 80%. At the final 5650th iteration (50th epoch), the value of mini-batch loss dropped to 0.036.

### 2.3. HLT-Based Bolt Angle Monitoring

#### 2.3.1. Image Correction of Perspective Distortion

Homography is a projective adjusting method that allows rectifying a distorted image based on a homography matrix *H*. The primary concept is to transform a point *a_j_* = (*u_j_*, *v_j_*, 1) in an image plane into a matching point *b_j_* = (*x_j_*, *y_j_*, 1) in another global plane by using the homography matrix *H* as below:

(1) $$a_{j}=Hb_{j},\;\mathrm{where}\;H=\left[\begin{array}{ccc} h_{11} & h_{12} & h_{13}\\ h_{21} & h_{22} & h_{23}\\ h_{31} & h_{32} & 1 \end{array}\right]$$

in which the matrix *H* has eight degrees of freedom (DOFs) which are 3 DOFs in translation, 3 DOFs in rotation, and 2 DOFs in scaling transformation.

**Figure 7:**
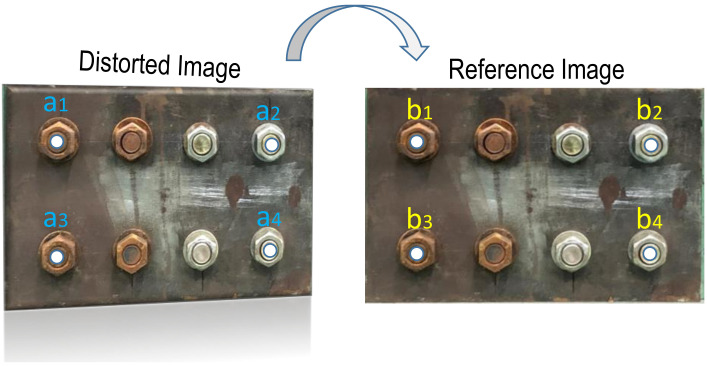
Image correction of perspective distortion.

As formulated in Equation (2), four-points pairs of the distorted image and their corresponding pairs of the reference image, *a_j_* and *b_j_* (*j* = 1:4), are used to calculate the homography matrix *H*. The known four points in the distorted image, *a_j_* (*j* = 1:4), are four corner bolts which are detected by the RCNN-based deep learning model. The corresponding four points in the reference plane, *b_j_* (*j* = 1:4), are identified by measuring the real distance of four corner bolts in the real plate, as illustrated in Figure 7.

(2) $$\left(\begin{array}{cccccccc} x_{1} & y_{1} & 1 & 0 & 0 & 0 & {-u}_{1}x_{1} & {-u}_{1}y_{1}\\ 0 & 0 & 0 & x_{1} & y_{1} & 1 & {-v}_{1}x_{1} & {-v}_{1}y_{1}\\ x_{2} & y_{2} & 1 & 0 & 0 & 0 & {-u}_{2}x_{2} & {-u}_{2}y_{2}\\ 0 & 0 & 0 & x_{2} & y_{2} & 1 & {-v}_{2}x_{2} & {-v}_{2}y_{2}\\ x_{3} & y_{3} & 1 & 0 & 0 & 0 & {-u}_{3}x_{3} & {-u}_{3}y_{3}\\ 0 & 0 & 0 & x_{3} & y_{3} & 1 & {-v}_{3}x_{3} & {-v}_{3}y_{3}\\ x_{4} & y_{4} & 1 & 0 & 0 & 0 & {-u}_{4}x_{4} & {-u}_{4}y_{4}\\ 0 & 0 & 0 & x_{4} & y_{4} & 1 & {-v}_{4}x_{4} & {-v}_{4}y_{4} \end{array}\right)\left[\begin{array}{c} h_{11}\\ h_{12}\\ h_{13}\\ h_{21}\\ h_{22}\\ h_{23}\\ h_{31}\\ h_{32} \end{array}\right]=\left\{\begin{array}{c} u_{1}\\ v_{1}\\ u_{2}\\ v_{2}\\ u_{3}\\ v_{3}\\ u_{4}\\ v_{4} \end{array}\right\}$$

#### 2.3.2. HLT-Based Bolt-Loosening Estimation

The HLT-based bolt angle estimation consists of five main steps: (1) to extract a correct bolt image from the bolt detector, (2) to apply a canny edge detector [18] for recognizing nut edges on the extracted bolt image, (3) to localize the detected nut edges of the image into Hough space, (4) to define a rough threshold for the extracted lines in Hough space, and (5) to draw the extracted lines in the bolt nut image.

**Figure 8:**
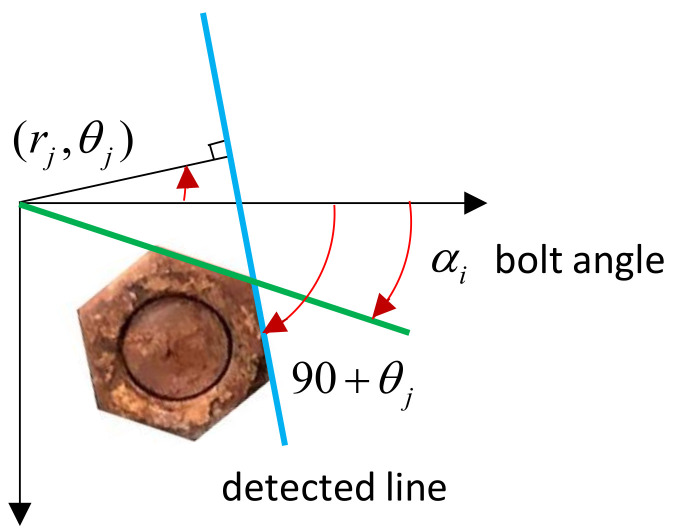
HLT-based bolt angle estimation.

Figure 8 depicts HLT-based bolt angle estimation. The position of the detected line (i.e., the blue line) is identified by a couple of parameters (*r_j_,*, *θ_j_*). The parameter *r_j_,* is the distance of the extracted line from Hough space to the origin of the coordinates axis, and *θ* is the rotational angle of the detected line. Then the *j*–th element position of the detected line can be expressed by two parameters ($r_{j}$,$\theta_{j}$), where $r_{j}\geq 0$, and $\theta_{j}\in [-\pi ,\pi ]$:

(3) r=xcosθ+ysinθ

As illustrated in Figure 8, the bolt angle (*α_j_*) is defined as the combination between horizontal coordinates axis and the green line. The bolt angle in the range of 0°–60° is calculated as the remainder of modulo operation as described in Equation (4). As defined in Equation (5), the final bolt angle is considered as the average angle of the *j*–th detected line element, where *k* holds values of 1–6 corresponding to a hexagon edge.

(4) $$\alpha_{j}=\mathrm{mod}[(\theta_{j}+90)/60]$$

(5) $$\alpha =\frac{1}{k}\sum_{j=1}^{k}\alpha_{j}$$

#### 2.3.3. Damage Classification based on Upper Control Limit (UCL)

The angle of the loosened bolt is identified by comparing the rotational angle value α of the initial bolt (i.e., the intact case) with the current rotational angle $\alpha^{*}$ of the damaged state, as shown in Equation (6). The absolute value of the detected loosening angle is used to compare with the threshold definition of *UCL* of Equation (7). Bolts are identified as the loosened bolts if the absolute value of Δα is greater than the *UCL* threshold; otherwise, no loosening. The *UCL* threshold is computed by three standard deviations of the mean, as shown in Equation (8), which corresponds to the confidence level of 99.7%.

(6) $$\Delta \alpha =\alpha^{*}-\alpha$$

(7) $$\left|\Delta \alpha \right|>UCL$$

(8) UCL=μ+3σ

## 3. Lab-Scale Experimental Setup

**Figure 9:**
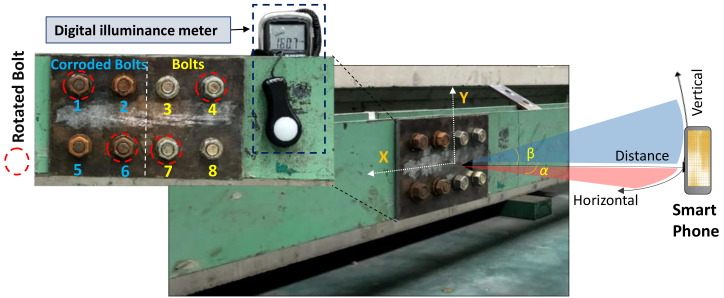
Experimental setup for bolt detection in steel girder connection.

The feasibility of the proposed method was experimentally evaluated in a bolted connection of a steel girder. As shown in Figure 9, an H-shape steel girder was built with a bolted connection in the center. Eight pairs of bolts and nuts (ϕ20 Korean standard) and a couple of splice plates (310 × 200 ×10 mm) were divided into two sides of the steel girder. For the comparative evaluation, four artificially corroded bolts and four non-corroded clean bolts were placed on the left-hand and right-hand sides, separately. Artificial corrosion of the samples (i.e., bolts and nuts) was generated in wet conditions coupled with an acid liquid (10% HCl).

The dual cameras of an iPhone X were used to capture the photos of the target steel connection. The camera has several functions including 12 megapixels, wide-angle f/1.8 aperture, telephoto f/2.4 aperture, and HEIF and JPEG image formats with 3042 × 4028 pixel resolutions. The lab-scale tests were repeated three times to assure the performance of the proposed bolt-lessening detection method.

**Figure 10:**
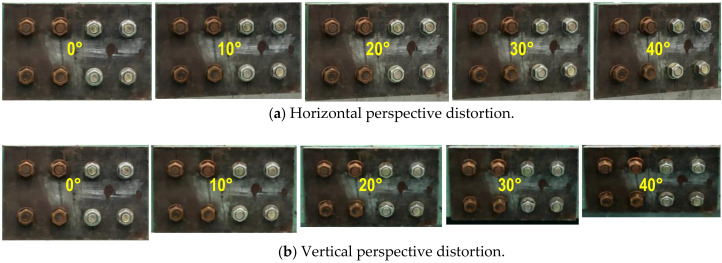
Testing images for various perspective distortions.

**Figure 11:**
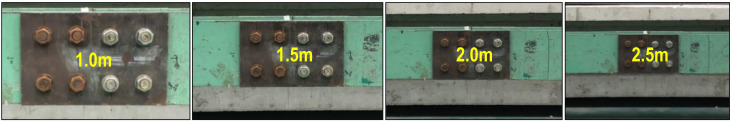
Testing images for different capture distances.

**Figure 12:**
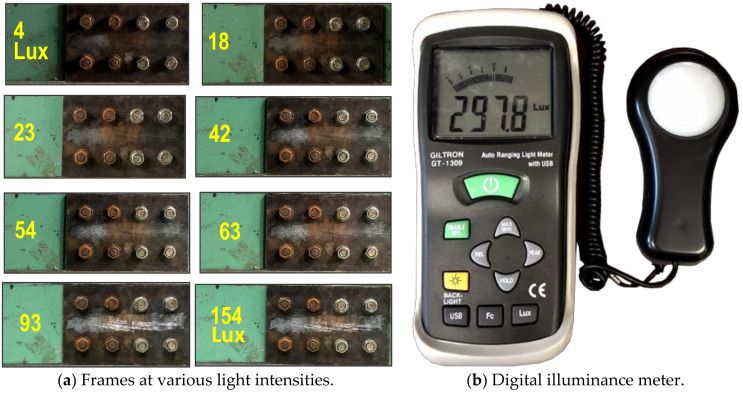
Testing images and measuring equipment for various light intensities.

For corrosion damage, three uncertain conditions were tested to evaluate the accuracy of the bolt detector, as follows: perspective distortion angles, image capture distances, and light intensities. Firstly, several capture angles (i.e., α, β = 0–40°) were taken with respect to the horizontal and vertical directions to examine the effect of perspective distortion on the proposed bolt detection model. As shown in Figure 10, total 90 images were captured corresponding to 9 horizontal and vertical distortion angles (i.e., 10 images for each angle). Next, several capture distances (1.0–2.5 m) were simulated to estimate their effects on the bolt detection model. As shown in Figure 11, a total of 40 images were shot for the four capture distances (i.e., 10 images for each distance). The perspective angle was kept as 0° and the level of light intensity was set as approximately 154 lux. Finally, various light intensities were simulated to estimate their effects on the accuracy of the bolt detector. In order to evaluate the effect of light intensity on the proposed framework, a digital light meter (GT 1309, GILTRON, Seoul, Korea) was placed at a girder connection nearby to measure the luminance from different electric light sources. As illustrated in Figure 12a, the image varied as the light intensity varied 4 lux to 154 lux. A total of 80 images were taken for eight different cases in the range of roughly 4.2 lux–154 lux. The capture distance was fixed as 1.5 m, and the perspective angle of the camera remained consistently at 0°.

Besides corrosion damage, four out of eight bolts were set up as bolt-loosening examples as shown in Figure 9. Two rusted bolts (i.e., bolts 1 and 6) were released by angles of 6° and 13°, respectively. The other two non-corroded bolts (i.e., bolts 4 and 7) were released by 10° and 26°. To simulate the damage cases, the corroded bolts 1 and 6 were released by 6° and 13°, respectively. Also, the non-corroded bolts 4 and 7 were released by 10° and 26°, respectively. The total 20 images were captured for the test, in which the first 10 images were captured for intact bolts (i.e., un-rotated bolts) and the other 10 images (11th–20th) were captured for the damage cases (i.e., rotated bolts).

## 4. Experimental Evaluation of the Bolt Monitoring Framework

### 4.1. RCNN-Based Bolt Detection under Uncertain Conditions

#### 4.1.1. Bolt Identification under Various Perspective Distortions

**Figure 13:**
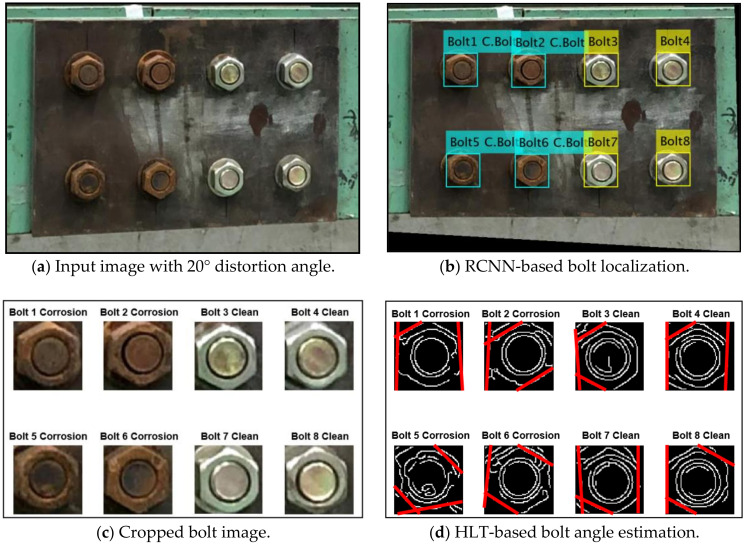
Bolt detection and angle estimation process for perspective distortion image.

Various capture angles were utilized to evaluate the effect of perspective distortion images on the accuracy of bolt detection by the proposed monitoring framework. In totally five capture-angles (0°, 10°, 20°, 30°, and 40°) were selected to simulate the horizontal and vertical distortion images, respectively. Figure 13 shows a process of bolt detection and angle estimation for a perspective distortion image. As shown in Figure 13a, a representative input image was tested for the horizontal distortion angle of 20°. Next, the projective transformation algorithm was applied to well rectify the distortive image (see Figure 13b). Then, the two types (i.e., corroded and non-corroded) of bolts were accurately localized from the trained RCNN-based model. The localized bolts were numbered to crop out into individual bolt images (see Figure 13c). On the basis of each cropped image, bolt angles were estimated using the HLT algorithm. Figure 13d shows the estimated bolt angles in the girder connection after the perspective correction.

**Figure 14:**
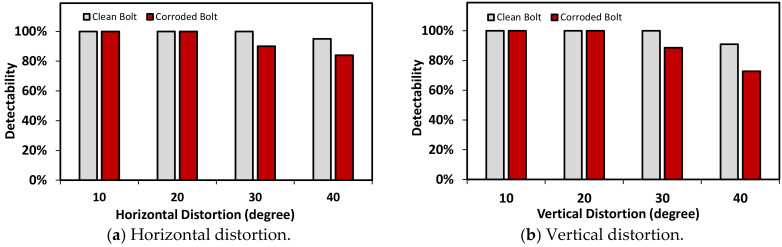
Effect of perspective distortion on the accuracy of bolt detector.

As shown in Figure 14, the effect of perspective distortion on the accuracy of the bolt detector was estimated for the five horizontal distortion cases (see Figure 14a) and also the five vertical distortion cases (Figure 14b). The accuracy of the bolt detector was high at the two distortion angles 10° and 20° for all bolt types (i.e., two corroded bolts and two clean ones). The detectability was decreased as the distortion angle was increased up to 40°. The non-corroded clean bolts were relatively well identified as 95% detectability for both horizontal and vertical distortions. The corroded bolts were less accurately identified by 75%~85% detectability (75% for vertical distortion and 85% for horizontal distortion). The dark colors of the rusted bolts led the difficulty in the bolt detection as the perspective angle increased.

#### 4.1.2. Bolt Identification under Various Capture Distances

**Figure 15:**
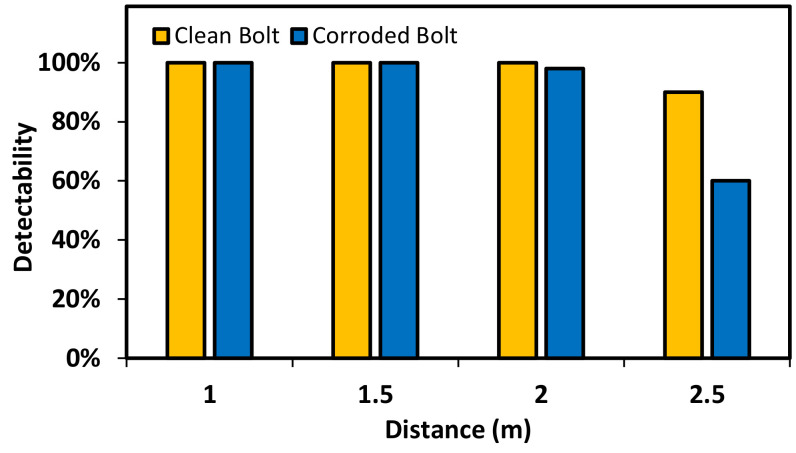
Effect of capture distance on the accuracy of bolt detector.

The effect of capture distance on the bolt detector was experimentally analyzed as shown in Figure 15. The bolt detection was accurate for the image captured at the distance less than 2.5 m. The detectability of bolt detection was accurate for the clean bolt and 95% for the corroded bolt by using the image captured at 2 m. A significant reduction of the accuracy was observed if the shooting distance increased up to 2.5 m.

#### 4.1.3. Bolt Identification under Various Light Intensities

**Figure 16:**
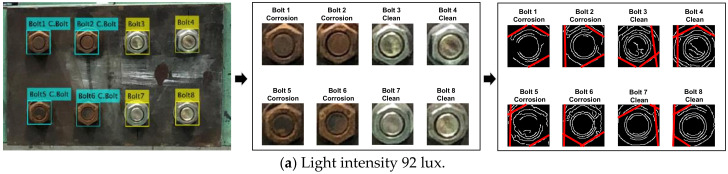

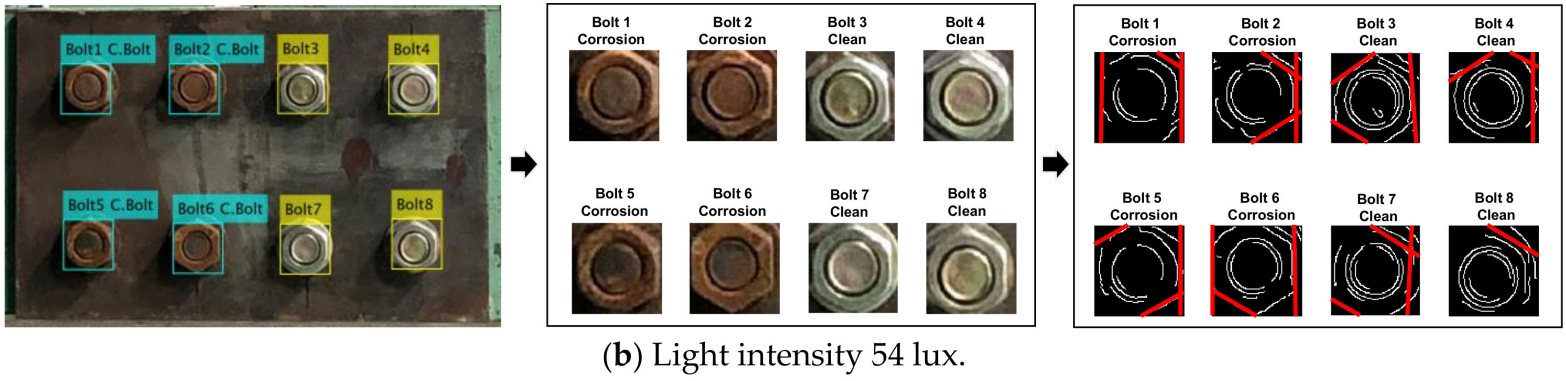
Bolt detection and contour identification results for various light intensities.

**Figure 17:**
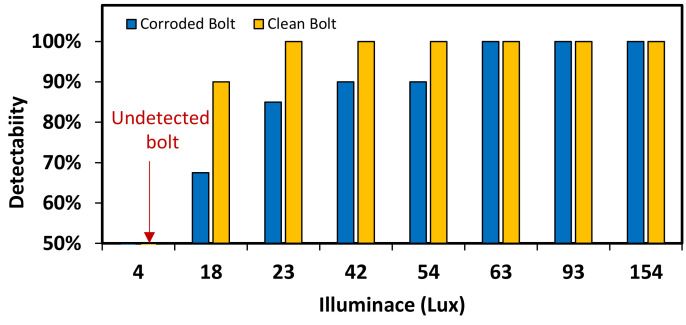
Effect of light intensity on the accuracy of the bolt detector.

Figure 16 shows the results of bolt detection and contour identification for two light intensities. As shown in Figure 16a, the operation of bolt detection and angle identification was successful for both clean and corroded bolts at 92 lux. As shown in Figure 16b, the operation was partially successful for corroded bolts at 54 lux. The effect of light intensity on the accuracy of bolt detection and contour identification was analyzed as shown in Figure 17. For the corroded bolt, the detectability of bolt detection decreased to roughly 90% at the light intensity of 54 lux, as shown in Figure 17. For the clean bolt, the same 90% detectability was resulted at 18 lux. Due to the change of light intensity, the change of bolt contour was more affected in the rusted bolt rather than the clean bolt.

### 4.2. HLT-Based Bolt Angle Estimation

#### 4.2.1. Bolt-Loosening Estimation under Various Perspective Distortion

**Figure 18:**
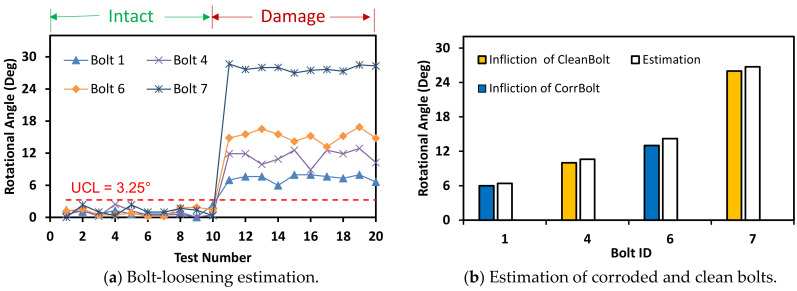
Bolt-loosening detection at distorted image of 10°.

A horizontally distorted image at 10° was tested to evaluate the bolt-loosening estimation, as shown in Figure 18. The bolt angles were estimated with a threshold UCL = 3.25°. The estimated bolt angles of the damage case (i.e., 10th–20th images) were classified as beyond the UCL threshold. The sensitivity of HLT-based bolt-loosening detection results was compared for the corroded and clean bolts, as shown in Figure 18b. It is observed that the proposed bolt detection method was consistent for both corroded and clean bolts.

**Figure 19:**
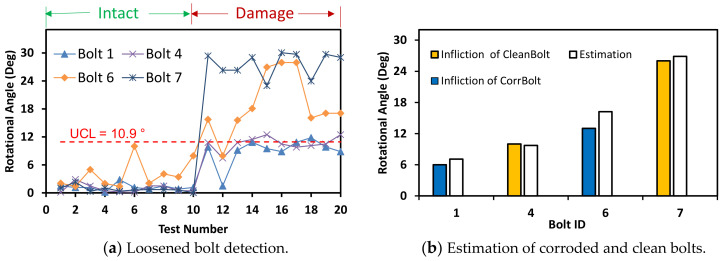
Bolt-loosening detection at distorted image of 30°.

**Figure 20:**
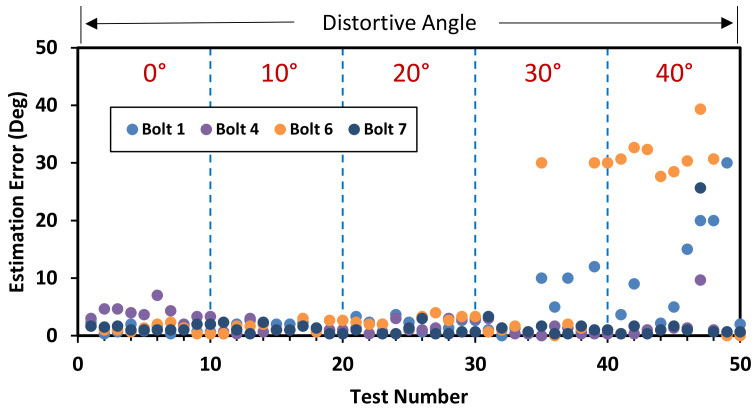
Bolt angle estimation error with respect to perspective distortion angles.

Also, another horizontally distorted image of 30° was tested similarly as shown in Figure 19. The bolt angles were estimated with a threshold UCL = 10.9°. Although the accuracy was a little reduced due to the perspective angle, the estimated angles of the bolts were classified beyond the UCL threshold. Figure 20 shows the bolt angle estimation errors with respect to the perspective distortion angles for the corroded and clean bolts. For 0°–20° of perspective distortion, the bolt angle estimation errors were steady small in all corroded and clean bolts. For 30°–40° of the perspective distortion, the estimation errors were relatively small in clean bolts 4 and 7 but relatively high in corroded bolts 1 and 6. It is observed that the HLT-based bolt angle estimation was accurate in the corroded and clean bolts.

#### 4.2.2. Bolt-Loosening Estimation under Various Image Capture Distances

**Figure 21:**
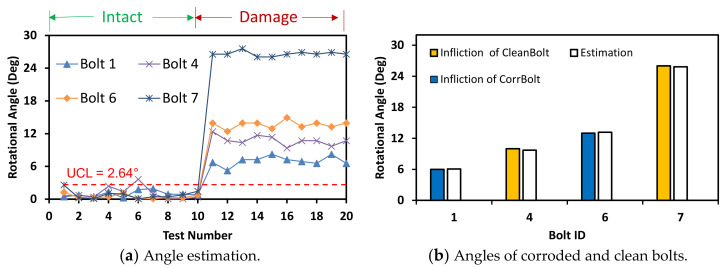
Bolt-loosening estimation for image capture distance of 1 m.

**Figure 22:**
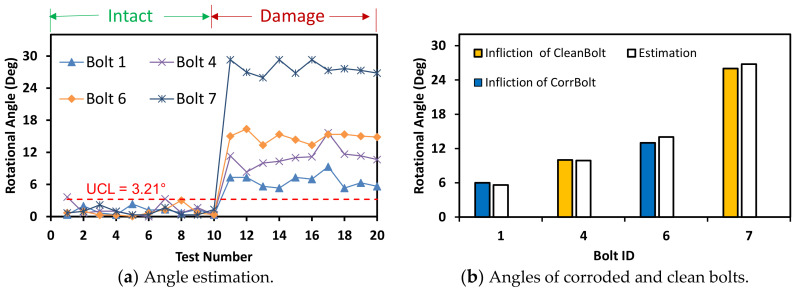
Bolt-loosening estimation for image capture distance of 1.5 m.

The accuracy of bolt-loosening estimation was quantified with respect to the four image capture distances between 0 m and 2.5 m. For the capture distance of 1.0 m, the bolt angles were accurately estimated for the corroded and clean bolts with a threshold UCL = 2.64° (see Figure 21). For the capture distance of 1.5 m, the bolt angles were accurately estimated for the corroded and clean bolts with a threshold UCL = 3.21° (see Figure 22). For the intact case (i.e., no bolt-loosening case), most of bolt angles, which were estimated from the first 10 images, were below the UCL thresholds. For the bolt-loosening cases, bolt angles estimated from the 11th–20th images were beyond the UCL thresholds, well indicating the occurrence of damage in bolts 1, 4, 6, and 7. The accuracy of bolt-loosening estimation was relatively high for all bolts, as shown in Figure 21b and Figure 22b.

**Figure 23:**
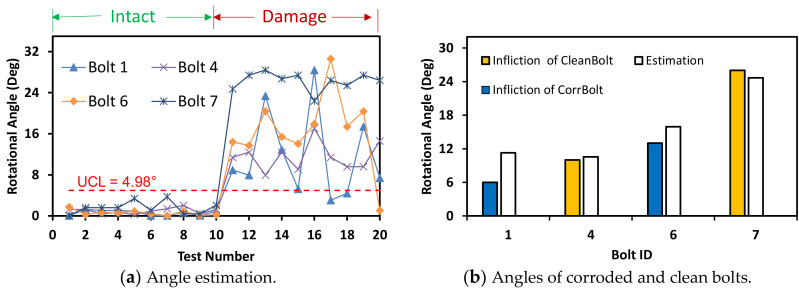
Bolt-loosening estimation for image capture distance of 2 m.

The accuracy of bolt angle estimation was reduced as the shooting distance increased above 2.0 m. As shown in Figure 23, the threshold became UCL = 4.98° for the capture distance of 2.0 m producing relatively overestimation for the corroded bolts. A significant change in bolt-loosening detection occurred by the shooting distance of 2.0 m. Some of the corroded bolts 1 and 6 were recognized as unloosened ones for both intact and damage cases; meanwhile, all of the clean bolts 4 and 7 were well detected for both cases.

#### 4.2.3. Bolt-loosening Estimation under Various Light Intensities

**Figure 24:**
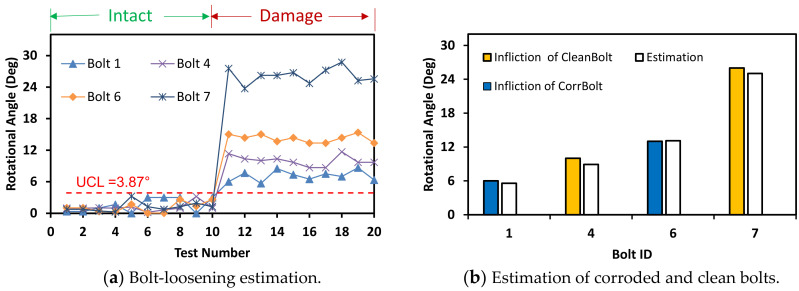
Bolt-loosening estimation for light intensity of 93 lux.

**Figure 25:**
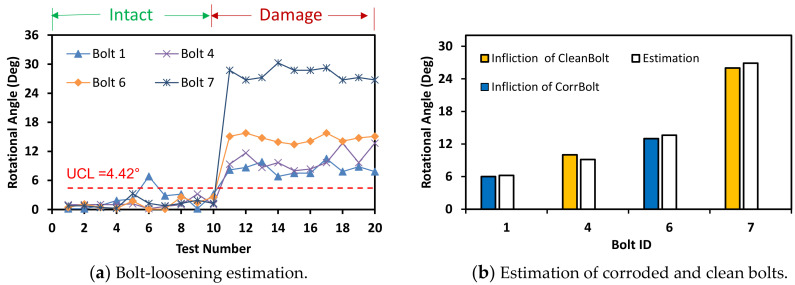
Bolt-loosening estimation for light intensity of 63 lux.

The accuracy of bolt-loosening estimation was evaluated for the three different light intensities. For the light intensity of 93 lux, as shown in Figure 24, the accuracy of bolt angle detection was guaranteed by the UCL = 3.87°. All bolts 1, 4, 6, and 7 were accurately recognized for the intact and the damage cases. For the light intensity of 63 lux, as shown in Figure 25, the bolt detection was quantified by the UCL = 4.42°. Most of the bolts in the damage cases were higher than UCL, indicating the occurrence of damage.

**Figure 26:**
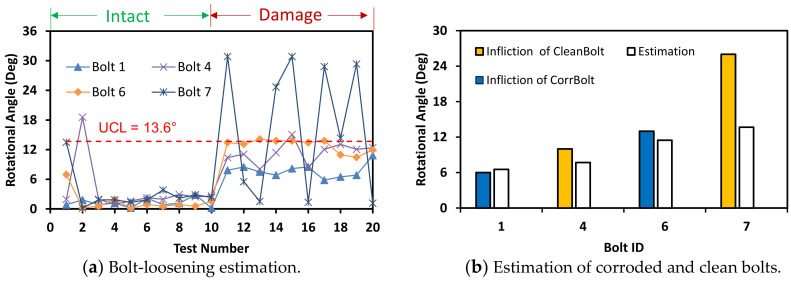
Bolt-loosening estimation for light intensity of 54 lux.

Next, the light intensity was decreased to 54 lux, as shown in Figure 26. The accuracy of bolt detection was guaranteed by the increased UCL = 13.6°. As shown in Figure 26a, bolts 1, 4, and 6 were classified as the loosened bolts, while the bolt 7 fluctuated around the threshold. It is observed that the light intensity below about 60 lux might result in inaccurate estimation of bolt-loosening.

## 5. Conclusions

In this study, a two-phase framework which orderly applied the regional convolutional neural network (RCNN)-based deep learning and Hough line transform (HLT) algorithms was presented to identify corroded bolts and also to estimate bolt-loosening. Lab-scale experiments were performed under three uncertain conditions (i.e., angle distortion, capture distance, and light intensity) to evaluate the feasibility of the RCNN-based bolt detector and HLT-based bolt angle estimation.

From the results, at least three major observations can be made as follows:

(1) The proposed RCNN-based deep learning method could accurately identify rusted bolts distinguished from clean ones under the perspective distortion less than 15°, the image capture distance less than 1.5 m, and the light intensity larger than 63 lux.
(2) The HLT-based method could accurately detect a loosened bolt with small incipient rotation of 3.25° by using images captured under the perspective angle equal to or less than 10°, the capture distance of 1 m, and the light intensity of 93 lux. However, the accuracy of bolt angle estimation was significantly decreased under highly-distorted perspective angles (i.e., more than 20°), long-distanced captures (i.e., more than 2 m), and low-intensity lights (i.e., less than 54 lux).
(3) The damage detection (i.e., bolt-loosening monitoring) of the corroded bolts was more difficult than the non-corroded bolts, which might be due to the effect of pollutants and dirt sticking on the edges of the corroded bolts. Also, the intensity of the stacked corrosion on the bolt surface changed over time, which resulted in the gradual decrease in brightness level of rust color and consequently caused the difficulty in identifying the corroded bolts.

The current framework could distinguish fast-rusted bolts from the clean bolts. The proposed method could be applied for multiple damage types like corrosion and bolt-loosening in a steel structure by using a single frame of image. For practical applications, the proposed study can be implemented to build training data for recognizing real environment-induced corroded bolts. Also, the proposed methodology can be integrated with unmanned aerial vehicles to provide timely warnings in detecting quick-rusted bolts and bolt-loosening in steel structures in hazardous environmental conditions under designed light intensity.

Future studies will assess the influence of time-dependent image-gathering on the performance of training images. In reality, bolts often start to leak partially before spreading out to the entire bolted connection; therefore, adding more image classes to train partially rusted bolts will ensure the accuracy of bolt identification. Also, a further study should be made to monitor rust color pixels on bolt surfaces by adding the branch of fully connected network (FCN).

## Figures and Tables

**Table 1:** Details on CNN architecture based on AlexNet [26].

No.	Layer	Size	Operator	Filter Size	Number	Stride	Padding
1	L1	227 × 227 × 3	Input	-	-	-	-
2	L2	55 × 55 × 96	Conv1	11 × 11 × 3	96	4	0
	L3	55 × 55 × 96	ReLU1	-	-	-	-
	L4	55 × 55 × 96	Norm1	-	-	-	-
	L5	27 × 27 × 96	MaxPooling1	3 × 3		2	0
3	L6	27 × 27 × 256	Conv2	5 × 5 × 48	256	1	2
	L7	27 × 27 × 256	ReLU2	-	-	-	-
	L8	27 × 27 × 256	Norm2	5 × 5 × 3	64	1	2
	L9	13 × 13 × 256	MaxPooling2	3 × 3		2	0
4	L10	13 × 13 × 384	Conv3	3 × 3 × 256	384	1	1
	L11	13 × 13 × 384	ReLU3	-	-	-	-
5	L12	13 × 13 × 384	Conv4	3 × 3 × 192	384	1	1
	L13	13 × 13 × 384	ReLU4	-	-	-	-
6	L14	13 × 13 × 256	Conv5	3 × 3 × 192	256	1	1
	L15	13 × 13 × 256	ReLU5	-	-	-	-
	L16	6 × 6 × 256	MaxPooling3	3 × 3		2	0
7	L17	1 × 1 × 4096	FCLayer6	1 × 1 × 4096	9216	-	-
	L18	1 × 1 × 4096	ReLU6	-	-	-	-
	L19	1 × 1 × 4096	Dropout Layer6	-	-	-	-
8	L20	1 × 1 × 4096	FCLayer7	1 × 1 × 4096	4096	-	-
	L21	1 × 1 × 4096	ReLU7	-	-	-	-
	L22	1 × 1 × 4096	Dropout Layer7	-	-	-	-
9	L23	1 × 1 × 3	FCLayer8	1 × 1 × 3	3	-	-
	L24	1 × 1 × 3	Softmax Layer	-	-	-	-
10	L25	1 × 1 × 3	Output	-	-	-	-

## References

[1] Wang T., Song G., Liu S., Li Y., Xiao H. (2013). Review of Bolted Connection Monitoring. Int. J. Distrib. Sens. Netw..

[2] Nikravesh S.M.Y., Goudarzi M. (2017). A Review Paper on Looseness Detection Methods in Bolted Structures. Lat. Am. J. Solids Struct..

[3] Pidaparti R.M. (2016). Structural Corrosion Health Assessment using Computational Intelligence Methods. Struct. Health Monit. Int. J..

[4] Ye X.W., Dong C.Z., Liu T. (2016). A Review of Machine Vision-Based Structural Health Monitoring: Methodologies and Applications. J. Sens..

[5] Spencer B.F., Hoskere V., Narazaki Y. (2019). Advances in Computer Vision-Based Civil Infrastructure Inspection and Monitoring. Engineering.

[6] Sun L., Shang Z., Xia Y., Bhowmick S., Nagarajaiah S. (2020). Review of Bridge Structural Health Monitoring Aided by Big Data and Artificial Intelligence: From Condition Assessment to Damage Detection. J. Struct. Eng..

[7] Yang J., Chang F.-K. (2006). Detection of bolt loosening in C–C composite thermal protection panels: II Experimental verification. Smart Mater. Struct..

[8] Blachowski B., Swiercz A., Pnevmatikos N. Experimental verification of damage location techniques for frame structures assembled using bolted connections. Proceedings of the 5th International Conference on Computational Methods in Structural Dynamics and Earthquake Engineering.

[9] Huynh T.-C., Dang N.-L., Kim J.-T. (2017). Advances and Challenges in impedance-based structural health monitoring. Struct. Monit. Maint..

[10] Huynh T.-C., Kim J.-T. (2018). RBFN-based temperature compensation method for impedance monitoring in prestressed tendon anchorage. Struct. Control. Health Monit..

[11] Huynh T.-C., Kim J.-T. (2017). Quantification of temperature effect on impedance monitoring via PZT interface for prestressed tendon anchorage. Smart Mater. Struct..

[12] Park J.-H., Huynh T.-C., Choi S.-H., Kim J.-T. (2015). Vision-based technique for bolt-loosening detection in wind turbine tower. Wind Struct..

[13] Cha Y.-J., You K., Choi W. (2016). Vision-based detection of loosened bolts using the Hough transform and support vector machines. Autom. Constr..

[14] Nguyen T.-C., Huynh T.-C., Ryu J.-Y., Park J.-H., Kim J.-T. Bolt-loosening identification of bolt connections by vision image-based technique. Proceedings of the Nondestructive Characterization and Monitoring of Advanced Materials, Aerospace, and Civil Infrastructure 2016.

[15] Kong X., Li J. (2018). Image Registration-Based Bolt Loosening Detection of Steel Joints. Sensors (Basel).

[16] Ramana L., Choi W., Cha Y.-J. (2018). Fully automated vision-based loosened bolt detection using the Viola–Jones algorithm. Struct. Health Monit..

[17] Duda R.O., Hart P.E. (1971). Use of the Hough transformation to detect lines and curves in pictures. Commun. Acm..

[18] Canny J. (1986). A computational approach to edge detection. IEEE Trans. Pattern Anal. Mach. Intell..

[19] Gu J., Wang Z., Kuen J., Ma L., Shahroudy A., Shuai B., Liu T., Wang X., Wang G., Cai J. (2015). Recent advances in convolutional neural networks. Pattern Recognit..

[20] Zhao X., Zhang Y., Wang N. (2019). Bolt loosening angle detection technology using deep learning. Struct. Control Health Monit..

[21] Atha D.J., Jahanshahi M.R. (2017). Evaluation of deep learning approaches based on convolutional neural networks for corrosion detection. Struct. Health Monit..

[22] Cha Y.-J., Choi W., Suh G., Mahmoudkhani S., Büyüköztürk O. (2018). Autonomous Structural Visual Inspection Using Region-Based Deep Learning for Detecting Multiple Damage Types. Comput. Aided Civ. Infrastruct. Eng..

[23] Huynh T.-C., Park J.-H., Jung H.-J., Kim J.-T. (2019). Quasi-autonomous bolt-loosening detection method using vision-based deep learning and image processing. Autom. Constr..

[24] Girshick R., Donahue J., Darrell T., Malik J. Rich feature hierarchies for accurate object detection and semantic segmentation. Proceedings of the IEEE Conference on Computer Vision and Pattern Recognition.

[25] Uijlings R.R.J., Van de Sande A.E.K., Gevers T., Smeulders M.W.A. (2012). Selective Search for Object Recognition. Int. J. Comput. Vis..

[26] Krizhevsky A., Sutskever I., Hinton E.G. (2012). ImageNet Classification with Deep Convolutional Neural Networks. Adv. Neural Inf. Process. Syst..

